# The Role of the Preanalytical Step for Human Saliva Analysis via Vibrational Spectroscopy

**DOI:** 10.3390/metabo13030393

**Published:** 2023-03-08

**Authors:** Beatrice Campanella, Stefano Legnaioli, Massimo Onor, Edoardo Benedetti, Emilia Bramanti

**Affiliations:** 1 Institute of Chemistry of Organometallic Compounds (ICCOM), Consiglio Nazionale delle Ricerche(CNR), 56124 Pisa, Italy; 2 Hematology Unit of Azienda Ospedaliero Universitaria Pisana (AOUP), 56100 Pisa, Italy

**Keywords:** saliva, ATR-FTIR, sample processing, Raman

## Abstract

Saliva is an easily sampled matrix containing a variety of biochemical information, which can be correlated with the individual health status. The fast, straightforward analysis of saliva by vibrational (ATR-FTIR and Raman) spectroscopy is a good premise for large-scale preclinical studies to aid translation into clinics. In this work, the effects of saliva collection (spitting/swab) and processing (two different deproteinization procedures) were explored by principal component analysis (PCA) of ATR-FTIR and Raman data and by investigating the effects on the main saliva metabolites by reversed-phase chromatography (RPC-HPLC-DAD). Our results show that, depending on the bioanalytical information needed, special care must be taken when saliva is collected with swabs because the polymeric material significantly interacts with some saliva components. Moreover, the analysis of saliva before and after deproteinization by FTIR and Raman spectroscopy allows to obtain complementary biological information.

## 1. Introduction

Saliva is a matrix rich of biochemical information. The term “*salivaomics*” was introduced in 2008 to indicate the complexity and the importance of knowing the various “omic” constituents of saliva (https://iadr.abstractarchives.com/abstract/2008Dallas-100600/salivaomics-knowledge-base-skb, accessed on 27 February 2023). It is quite clear that whole-mouth saliva contains a variety of high- (proteins and nucleic acids) or low-molecular-weight compounds (salts, organic and inorganic acids, sugars, and nitrogenous bases.) and that its analysis might disclose clinically relevant information regarding the oral and systemic health status [1,2] (and references therein). Saliva collection is noninvasive and straightforward; it has high patient compliance, and it can be easily repeated [3,4]. For this reason, many biological and bioanalytical techniques (chromatographic and spectroscopic) have been developed in the last 15 years to investigate *salivaomics* through targeted and untargeted methods [5,6].

Attenuated total reflectance-Fourier transformed infrared spectroscopy (ATR-FTIR) is a nondestructive/microdestructive, fast, and cost-effective spectroscopic approach that requires in principle minimal sample handling to collect information from biological samples, tissues, cells, or biofluids

Several reviews report on the application of mid-infrared (IR) as a promising tool in human saliva [2,7,8,9,10,11,12,13]. The analysis of saliva as a diagnostic specimen by ATR-FTIR in tandem with chemometric analysis has experienced a rapid growth over the last decade, and even more in the last 2–3 years. In 1996, a new quantitative method based on transmittance FTIR was developed to evaluate thiocyanate concentrations in 5 µL of dried human saliva [14] using the band at 2058 cm^−1^. More than 10 years later, Khaustova et al. developed an ATR-FTIR method to rapidly assess the biochemical properties of the saliva (total protein concentration, glucose, secretory immunoglobulin A, urea, amylase, cortisol, and inorganic phosphate) [15].

Recently, FTIR has been applied to study saliva from diabetic patients [16,17,18,19,20,21] and patients with oral pathologies [22,23] and to identify cancer biomarkers [4,24,25] and COVID-19-related biomarkers [26,27,28,29]. Recently, ATR-FTIR spectra in tandem with chemometric have been employed to analyze the spectral changes in semen, saliva, and urine in violent crimes during dry out, allowing to estimate their time since deposition [30].

Raman spectroscopy can yield complementary information to IR spectroscopy as the two techniques rely on different processes and selection rules. The inherently weak Raman signals of biological molecules, often overwhelmed by sample fluorescence, are counterbalanced by the fact that Raman spectra are mostly unaffected by water bands and exhibit sharper signals compared to IR. The application of Raman to saliva analysis was recently reviewed by Hardy et al. [31].

Although sample preparation in vibrational spectroscopy is minimal, several methodological features are critical to obtain reproducible, comparable spectra of saliva [8,13,32]. Thus, it is crucial to standardize the preanalytical step, including both saliva sampling [2,33] and sample preparation, to obtain time- and cost-effective procedures and to minimize sample handling and possible contaminations.

Saliva composition depends on the collection method, as well as on the nature and duration of salivation stimulation, subject hydration status, collection timing, etc. [2,33]. In many studies, vibrational spectra are acquired on dried samples, adopting a drying time variable between 3 min (directly drying the saliva sample onto the plate of the ATR device) and 24 h (after drying onto various supports for ATR and scattering analysis).

Table 1 summarizes the main works published in which FTIR spectroscopy was employed for the analysis of saliva, focusing on the brief descriptions of the preanalytical steps.

Basically, in all the works, the spectra were recorded on dried samples, i.e., after the removal of water. Water bands may indeed affect the sensitivity and reproducibility in the detection of several sample components, especially for IR.

In the last few years, our research group has extensively studied the salivary metabolites by liquid and gas chromatography approaches [45,46,47,48,49,50]. The analysis of saliva by ATR-FTIR and Raman provides complementary, fast, and holistic information on the sample, which includes low-molecular-weight (MW) metabolites and (macromolecules proteins, carbohydrates, and lipids), both having a high diagnostic value for local and systemic disorders.

The aim of this work is to investigate the effect of saliva sampling (spitting method or sampling with commercial polymeric swab) on the vibrational spectra (ATR-FTIR and Raman) acquired before and after deproteinization with two methods (protein precipitation with ethanol or using 3 kDa cut-off centrifugation units). The spitting method may indeed simplify the sampling, meeting patient compliance (especially for children) and reducing costs and risks. Saliva contains about 0.1–1.5 mg/mL protein [51], and the saliva deproteinization may simplify the spectral information, allowing the analyst to focus on the analytical window of interest. In all cases, information remains complex, and the coupling with chemometrics is crucial to extract information from the vibrational spectra. An easy “printing” of sample dried spots (SDSs) prepared on polypropylene (PP) sheets onto ATR crystal is described for the fast, interference-free acquisition of FTIR spectra.

Our work implements the information recently reported by Paschotto et al. [4], who investigated ATR-FTIR absorption of saliva sampled with different collection methods (spitting method vs. soaking) and processing protocols (dried unprocessed, dried supernatant after centrifugation, and dried concentrate), confirming the need of standardized collection–processing protocols based on the biochemical component analysis. Paschotto et al. investigated the effects of sampling using cotton swabs, and they applied centrifugation conditions at low *g* values, probably removing cells and bacteria. They did not investigate the deproteinization effect, nor were both FTIR and Raman spectroscopy used. In our work, the concentrations of the main metabolites in saliva after the various sample handling procedures were also determined by RP-HPLC-DAD [49] to focus on the possible artefacts of saliva sampling and sample handling.

## 2. Experimental Design

### 2.1. Chemicals

Sulfuric acid for HPLC analysis was employed (V800287 VETEC ≥ 85% Sigma-Aldrich, Milan, Italy). Methanol for RP-HPLC was purchased from Carlo Erba (Rodano, Italy). Preparation and dilution of samples and solutions were performed gravimetrically using ultrapure MilliQ water (18.2 MΩ cm^−1^ at 25 °C, Millipore, Bedford, MA, USA). Standard solutions for HPLC (TraceCERT^®^, 1000 mg/L in water) were purchased from Sigma-Aldrich, Milan, Italy. Analyte stock and working solutions were prepared as previously reported [49].

### 2.2. Experimental Design: Saliva Sample Collection and Processing

Whole, nonstimulated saliva samples were collected from 10 nominally healthy volunteers. The study was performed in accordance with the Declaration of Helsinki. Written informed consent was obtained from all volunteers who agreed to provide saliva samples. A fasting period of at least 8 h was required, and volunteers did not brush or rinse the oral cavity with mouthwash before sampling. Exclusion criteria included the existence of any oral disease or a systemic pathology, alcohol consumption, smokers, or systemic medication usage. The pattern of samples analyzed was the following: The volunteers were asked to spit into sterile polypropylene tubes (about 2 mL for each subject). Saliva samples were pooled, homogenized in vortex, and stored in a freezer at −20 °C. For the analysis, pooled saliva was thawed at room temperature and subdivided into two processing groups: one half (“salivette” in this work) was loaded onto Salivette^®^ swabs (2 mL/swab) for 5 min as physiological time for the adsorption of the whole saliva, centrifuged at 4500× *g* for 10 min at 4 °C (Eppendorf™ 5804R Centrifuge), and pooled again. Second half was used as is (unprocessed saliva, “saliva” in this work). This procedure was chosen to perform the methodological comparison exactly on the same sample, avoiding changes in saliva composition due to presence of the swab.

Both saliva and salivette samples were fractionated in three parts: (i) a part was analyzed as is (named saliva and salivette); (ii) a part was deproteinized by ultracentrifugation (30 min) using Microcon^®^ Centrifugal Filters with cut-off 3 kDa (Merk, Milan, Italy) (named saliva_CO and salivette_CO); (iii) a total of 100 μL of saliva or salivette was mixed with 900 μL ethanol (EtOH) (10-fold dilution), cooled at −20 °C for 2 h, and centrifuged at 14,000 rpm (10,000× *g*) for 30 min in a refrigerated centrifuge (named saliva_EtOH and salivette_EtOH). The solution remaining in the upper part of 3 kDa cut-off filtering units was also analyzed by ATR-FTIR to characterize the HMW compounds (“HMWsaliva_CO” and “HMWsalivette_CO”).

### 2.3. ATR-FTIR Analysis

**Figure 1:**
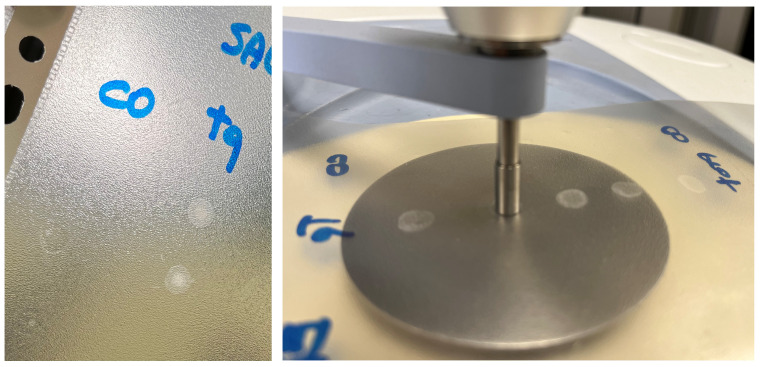
Saliva sample dried spot (SDS) from 50 μL deposition onto PP sheet and “printing” on ATR-FTIR crystal.

Five drops (50 μL each) of sample were deposited onto a polypropylene (PP) sheet by a micropipette (Eppendorf Research Plus pipette, Eppendorf AG) and air-dried at room temperature overnight. Spectra were recorded in ATR mode on sample dried spots (SDSs) using a Frontiers FTIR spectrometer (Perkin Elmer, Milan, Italy), equipped with a diamond-attenuated total reflectance (ATR) sampling accessory. The flat sample press tip (2 mm diameter) was employed to “stamp” the sample from the SDSs (Figure 1). After this, the PP sheet was removed. The microamount “printed” on the ATR diamond window was enough to obtain reliable and reproducible spectra. Using this method, at least 3 spectra can be recorded from 3 different areas of one single SDS. Spectra were recorded in 4000–600 cm^−1^ spectral range with a 4 cm^−1^ resolution, with 32 scans for the background and the sample. For each analysis, the diamond sampling window and the sample press tip were cleaned with 70% ethanol *v*/*v*. Mid-infrared (MIR) spectra were acquired on 3–5 different SDSs. Saliva_EtOH and salivette_EtOH sample spectra were acquired after the deposition of 3 μL of the samples directly onto the ATR crystal as ethanol evaporates in less than 15 s. HMWsaliva_CO and HMWsalivette_CO samples were analyzed by wiping (w) the tip wetted with the sample onto ATR crystal (samples dried in less than 15 s) or by “printing” (p) from SDSs.

### 2.4. Raman Analysis

Five drops (10 μL each) of sample were deposited onto a glass slide covered with an aluminum foil and air-dried at room temperature overnight. Spectra were recorded with a Renishaw inVia confocal micro-Raman system, coupled with an optical Leica DLML microscope equipped with an NPLAN objective 50×. The laser sources were a diode laser with a wavelength of 785 nm and an He–Ne laser with a wavelength of 633 nm. The spectrometer consisted of a single-grating monochromator (1200 or 1800 lines mm^−1^ according to the selected laser wavelength), coupled with a CCD detector, a RenCam 578 × 400 pixels (22 µm × 22 µm) cooled by a Peltier element. The spectral calibration of the instrument was performed on the 520.5 cm^−1^ band of a pure silicon crystal. Spectra were acquired with 633 nm laser source at 5.5 mW and with 785 nm laser source at 40 mW, 5 accumulations of 10 s each.

### 2.5. RP-HPLC-DAD Analysis

Saliva, salivette, saliva_CO, and salivette_CO samples were 5-fold diluted in 5 mM sulfuric acid, filtered using a 0.20 μm RC Mini-Uniprep (Agilent Technologies, Milan, Italy) filter, injected in the HPLC system (V_inj_ = 5 μL), and analyzed as previously reported [49]. Saliva_EtOH and salivette_EtOH were directly injected in the HPLC system (V_inj_ = 5 μL).

### 2.6. Data Processing

Principal component analysis (PCA) was carried out on the mean-centered column-wise spectra to investigate possible clustering of samples. ATR spectra were standardized by using standard normal variate (SNV) to minimize unwanted contributions (e.g., global intensity effects or baseline shifts). Raman spectra were treated to remove cosmic rays, and then Savitzky–Golay (zero-order derivative, third-degree polynomial order, and a window size equal to 9 data points) and Asymmetric Least Squares algorithms were applied for smoothing and baseline correction, respectively.

The analysis was performed with the open-source Chemometric Agile Tool (CAT) program (http://www.gruppochemiometria.it/index.php/software/19-download-the-r-based-chemometric-software, accessed on 27 February 2023) and by a tailored in-house R-script (R version 3.6.3 (R Development Core Team 2012) and R-Studio, Version 1.1.463) using the R-package mdatool.

## 3. Results and Discussion

### 3.1. ATR-FTR Analysis of Saliva/Salivette Dried Spots: Effect of Deproteinization Method

ATR-FTIR spectra were recorded on microspots “printed” from the dried spots on the ATR diamond window. The flat sample press tip (2 mm diameter) was employed to “stamp” the sample from the dried spots. After this, the PP sheet was removed. This procedure, not previously reported, allows in principle to prepare samples quickly onto a low-cost support and to obtain reliable and reproducible spectra using a microamount of sample. Using this method, at least three spectra can be recorded from three different areas of one single dried spot obtained from 50 μL.

**Figure 2:**
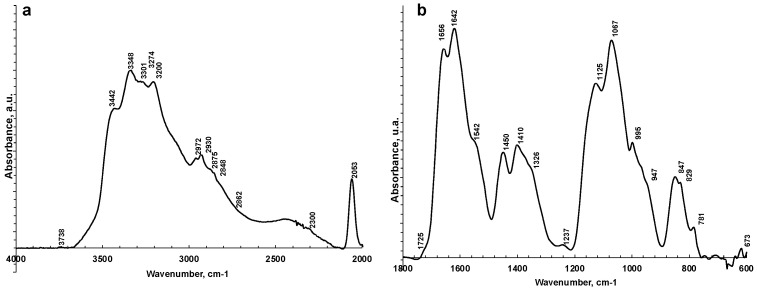
Representative ATR-FTIR spectra of saliva analyzed as is in 4000–2000 cm^−1^ (**a**) and 1800–600 cm^−1^ regions (**b**).

**Figure 3:**
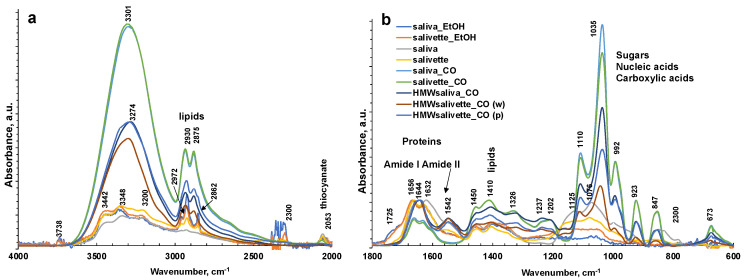

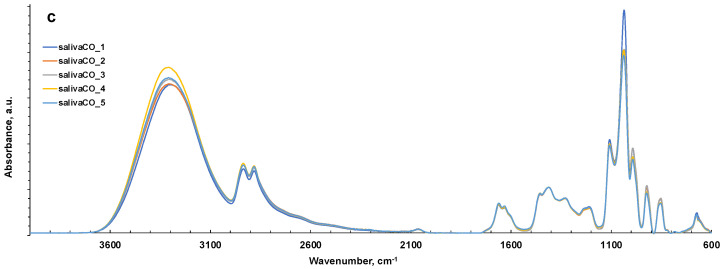
ATR-FTIR spectra of all representative saliva samples analyzed before and after deproteinization with ethanol (EtOH) and ultrafiltration with 3000 Da cut-off (CO) in 4000–2000 cm^−1^ (**a**) and 1800–600 cm^−1^ regions (**b**). (**c**) ATR-FTIR of N = 5 replicates of saliva sample after ultrafiltration with 3000 Da cut-off as example of reproducibility of the spectra. HMWsaliva_CO and HMWsalivette_CO refer to high-molecular-weight (HMW) compounds remaining in the upper part of 3 kDa cut-off filtering units (w = wiping, p = printing) as explained in the experimental part.

Figure 2 shows a representative ATR-FTIR spectrum of a saliva dried spot. Figure 3 shows the spectra of all the analyzed samples before and after SNV normalization. The absorption bands of lipids, proteins, carbohydrates, and nucleic acids are evidenced. The IR spectrum of saliva is in fact a superposition of the absorption spectra of all these components in proportion to their concentration, following the Lambert–Beer law.

The sampling and the deproteinization method employed evidenced major changes in the FTIR spectra of dried spots in the 1750–600 cm^−1^ fingerprint region and in the N–H and OH stretching regions (3800–1600 cm^−1^) and overlaid the latter in the region of C–H stretching in CH_2_ and CH_3_ (3000–2850 cm^−1^).

The FTIR spectrum of almost all samples examined showed the characteristic FTIR features of biological samples: the peaks of proteins at 1656–1642 cm^−1^ (Amide I, C=O stretching), 1542 cm^−1^ (Amide II, N–H bending), and 1237 cm^−1^ (Amide III); nucleic acids (1100–850 cm^−1^); P=O asymmetrical and symmetrical stretching vibrations of PO_2_ phosphodiester groups from phosphorylated molecules (1125 cm^−1^ and 1076 cm^−1^); and C–O stretching vibration coupled with C–O bending of the C–OH groups of carbohydrates (including glucose, fructose, and glycogen) at 1035 cm^−1^. The absorptions typical of proteins (Amide I, II, and III) were not observed in the saliva_CO and salivette_CO samples, i.e., after deproteinization by 3 kDa cut-off filtering. The spectral region 1080–950 cm^−1^ also includes the sugar moieties of glycosylated proteins, (e.g., salivary amylase and mucins). Several authors report the assignment of specific bands in the fingerprint region to immunoglobins (1560–1464 cm^−1^ associated to IgG, 1420–1289 cm^−1^ and 1160–1028 cm^−1^ related to IgM, and 1285–1237 cm^−1^ designed to IgA) [28]. However, the salivary proteome is a complex protein mixture resulting from the activity of salivary glands and serum, from mucosal and/or immune cells, or from micro-organisms containing amylase (representing about 20% of total proteins), mucins (about 20%), 6% human serum albumin, 10% lysozyme, 10% IgA and IgG, lactoferrin, proline-rich proteins, histatins, cathelicidins, defensins, glycoproteins, lipoproteins, statherin, and matrix metalloproteases [2,52,53]. Human saliva contains also inorganic compounds (sodium, potassium, calcium, magnesium, chloride, and phosphate) and organic nonprotein components, such as bilirubin, creatinine, glucose, lactic and uric acids [2], and references therein.

**Figure 4:**
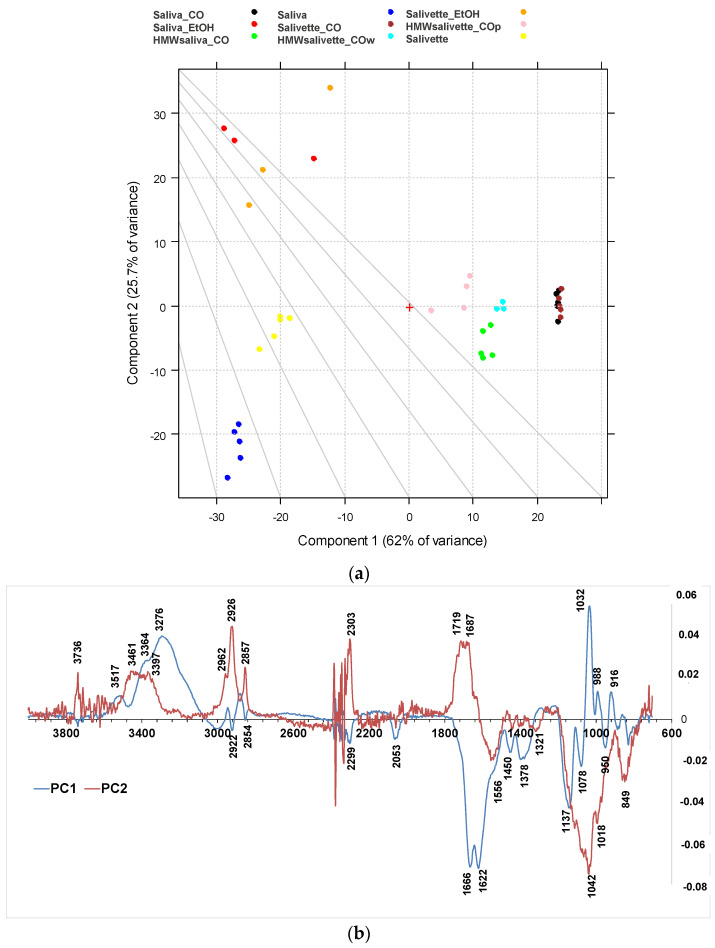
PCA results of SNV-normalized and centered ATR-FTIR spectra of saliva samples. (**a**) Score plot (87.7% of total variance); (**b**) loading plot of PC1 (blue line) and PC2 (red line). HMWsaliva_CO and HMWsalivette_CO refer to high-molecular-weight compounds (HMWCs) remaining in the upper part of 3 kDa cut-off filtering units (w = wiping, p = printing) as explained in the experimental part.

The differences among the various sample groups, corresponding to different saliva preparation modes, were better evidenced, and the information from the spectra were extracted using principal component analysis (PCA). The results derived from the PCA on the FTIR spectra are shown in the PC1–PC2 score plots (Figure 4a), explaining 87.8% of the total variance. PC1 is responsible for the separation of samples deproteinized using 3 kDa cut-off, which show positive values of PC1 (Figure 4b, blue line) with respect to the other samples on the left side of the plot. Interestingly, the HMWsaliva_CO and HMWsalivette_CO samples (MW > 3 kDa) cluster between unprocessed samples and saliva_CO/salivette_CO samples, without significant differences if analyzed by wiping the tip onto ATR crystal (w) or by “printing” from dried spots (p). PC2 (Figure 4b, red line) separates all samples treated with EtOH that show positive values of PC2 with respect to all the others. Figures S1 and S2 show the PC1–PC3 and PC2–PC3 scores (a) and loading plots (b), explaining 67.2% and 30.9% of the total variance, respectively.

The PC1 loading plot (Figure 4b, blue line) clearly shows positive values of 4000–3100 cm^−1^ absorptions related to OH and NH stretching vibrations, negative values of Amide I and Amide II bands typical of proteins due to C=O and C–N stretching vibrations, respectively, of the bands assigned to unsaturated C=CH stretching of lipids (at 3000 cm^−1^), symmetric -CH_3_ stretching at 2922 cm^−1^ due primarily to proteins, and symmetric -CH_2_ stretching at 2854 cm^−1^ due to lipids and proteins, and bending (at 1450 and 1378 cm^−1^) of the CH_2_ and CH_3_ groups. In the region of 3600–2900 cm^−1^, the absorption bands of the primary and secondary amines (-NH_2_ and -NHR) are observed; the peaks at 3300–3200 cm^−1^ are assigned to O–H vibrations; N–H stretching is typically around 3364–3517 cm^−1^ and usually show a medium, somewhat broad signal (usually considerably less broad than a typical OH stretching). The positive values of PC1 at 3200–3300 cm^−1^ reflect the higher contents of water in all saliva and salivette samples after deproteinization with 3 kDa units. Another important region of the FTIR spectrum is the spectral range 1180–800 cm^−1^ that originates from various C–C/C–O stretching vibrations in sugar moieties, P–O stretching of phosphate groups in phosphorylated proteins, and nucleic acids and low-MW compounds. The 1032 cm^−1^ band is usually attributed to the C–O stretching vibration in glycogen, while lactic acid has peaks at 1032 and 916 cm^−1^. Thus, the absorptions of low-MW metabolites in saliva/salivette spectra after 3 kDa cut-off ultrafiltration characterize PC1 components. The negative value in PC1, for these samples, of Amide I (1666–1622 cm^−1^) and Amide II bands (1556 cm^−1^), typical of proteins, also indicates that ultracentrifugation using 3 kDa cut-off is the only effective method for saliva deproteinization. The negative bands at 1137, 1078, 950, and 830 cm^−1^ of PC1 could be due to the removal of high-MW carbohydrates and nucleic acids from the saliva and salivette samples after cut off or the removal of phosphorylated molecules. The typical absorptions of high-MW compounds that characterize saliva and salivette samples are better evidenced in the negative components of PC3 (Figures S1 and S2, green line).

The PC2 loading plot shows remarkable positive values peaking at 3736, 3461, 3397 cm^−1^, 3022sh, 2962, 2926, 2878sh, and 2857 cm^−1^, characteristic of lipids. Positive values are also observed at 1750, 1719, and 1687 cm^−1^ and assigned to the C=O ester groups of lipids and cortisols and C=C stretching of cholesterol. These components are responsible for the clustering of the saliva_EtOH and salivette_EtOH samples. Among low-MW saliva components detected by FTIR, cortisol, phosphates, lactic acid, and urea are of interest from a medical point of view because their concentrations vary during physiological stress [44]. Our results suggest that the deproteinization in ethanol is not effective, in agreement with Araki, who reported that ethanol mostly precipitates non-protein nitrogen [54]. Table 2 shows with more detail the principal assignment of saliva MIR absorptions [7,10].

Negative values of the PC2 loading plot are observed at 1553, 1450, 1403, and 1321 cm^−1^. The differences between the saliva and salivette samples mainly rely on marked negative peaks of PC2 (Figure 4b), i.e., the absorptions at 1553 cm^−1^ (amide II), 1042 with shoulders at 1137 and 1018 cm^−1^, and 849 cm^−1^. These absorptions, typical of C–O–C symmetric and asymmetric vibrations of sugar moieties of heavily glycosylated proteins (e.g., mucins [31]) (Table 2), let us hypothesize that the polymeric swab (Salivette^®^) may adsorb proteins characterized by HMW and/or high degrees of glycosylation.

### 3.2. Choice of PP Support and Effect of Dried Spot Volume

Fifty μL was the optimized volume for the analysis of dried spots by FTIR that allowed to obtain “printed mini-spots” of suitable thickness to record high-quality FTIR spectra. If a smaller amount of sample is available for the analysis, e.g., 10 μL, the sample can be dried on PP and eventually gently scratched and microamounts analyzed by ATR-FTIR without significant changes in the spectra. The same experimental design performed on dried spots drop-casted onto aluminum foil did not gave satisfying, reproducible results likely because of the irregular thickness of the saliva dried spots or the rigidity of the aluminum foil. The good reproducibility of the saliva dried spots obtained on PP support may be also due to the hydrophobicity of the PP sheet itself. The ATR-FTIR measurements directly performed on the dried spots onto PP or aluminum foil have interference bands (data not shown for brevity) of the support employed unless higher volumes (≥50 µL) were used to obtain films of suitable thickness.

### 3.3. HPLC Analysis of Main Metabolites in Saliva/Salivette Samples

**Figure 5:**
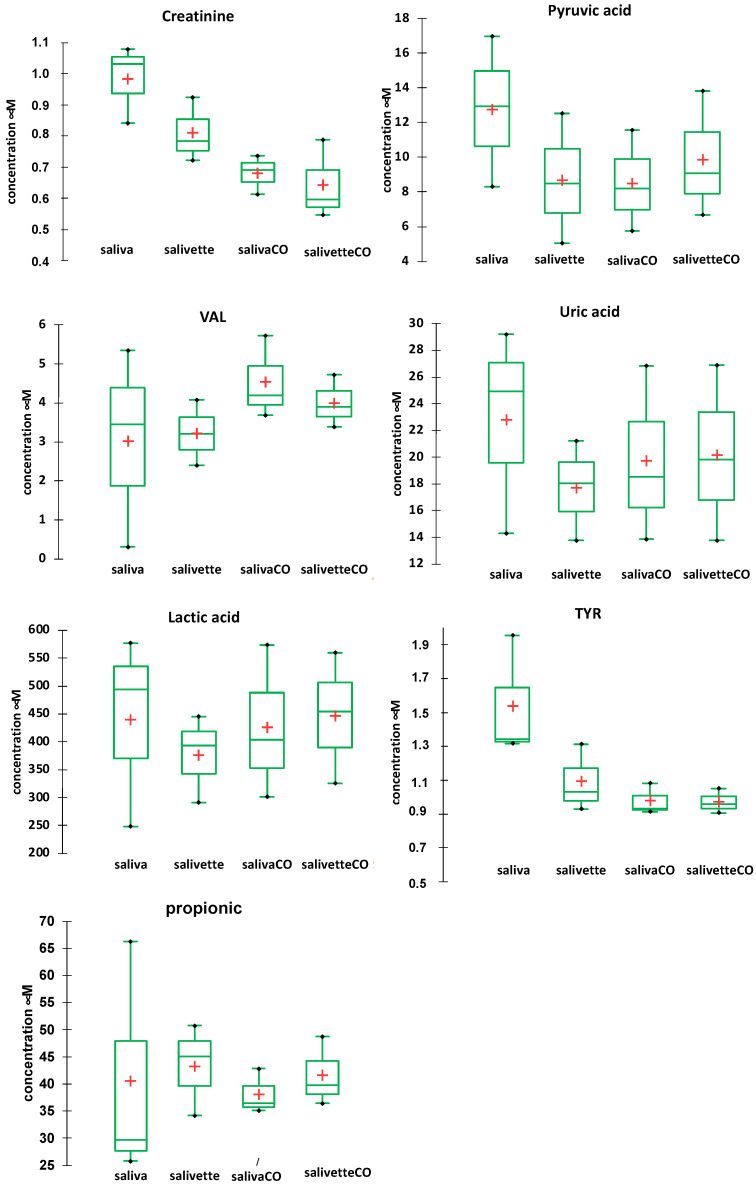
Box plots of the main metabolites determined in saliva/salivette samples before and after deproteinization with 3 kDa cut-off filtration. Red cross = mean value; black dots = minimum/maximum value; box = 1st quartile–3rd quartile range; bar = standard deviation.

The concentrations of the main metabolites in saliva after the various sample handling procedures were determined by RP-HPLC-DAD [49]. Figure 5 shows the comparison of the concentration (mean and SD) of seven main metabolites determined in the saliva/salivette samples before and after deproteinization with 3 kDa cut-off filtration. The injection of the saliva_EtOH and salivette_EtOH samples did not give meaningful results likely because the precipitation in ethanol favors reaction/degradation of LMW metabolites (e.g., the decrease in the peak of uric acid and the increase in an unassigned peak at t_R_ = 4.348 min) and the disappearance of the peaks of pyruvic acid, valine (VAL), lactic acid, and propionic acid (Figure S3a,b). Figure S3c shows, as an example, UV/visible spectra of the peak at t_R_ = 5269 min (orange line) of the saliva_CO sample, which is due to uric acid, and UV/visible spectra of the peaks at t_R_ = 5.2599 (purple line) and 4.35 min (blue line) of the saliva_EtOH sample. Both these peaks have the absorption characteristics of uric acid, but only the peak at 5.2599 has the same retention time of uric acid standard solution.

The results show that for most of the metabolites the sampling by spitting or by swab does not affect their quantitation (lactic, propionic, uric acids, and valine). For other metabolites (creatinine and pyruvic acid), the salivette swab seems to partially adsorb the analyte. The filtering with cut-off filtration units instead does not affect their quantitation.

### 3.4. Raman Analysis on Saliva Dried Spots

**Figure 6:**
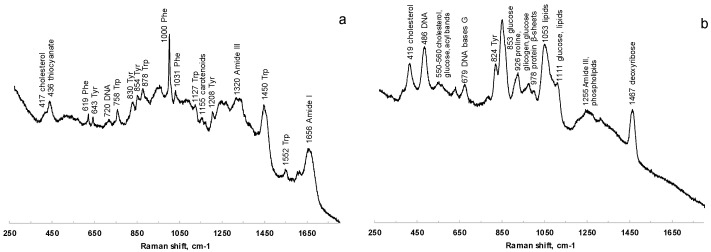
Comparison of Raman spectra at 785 nm of saliva before (**a**) and after (**b**) filtering with 3 kDa filters.

Raman spectra were acquired from saliva dried spots on PP, glass, and aluminum foil-covered glass. The signals of PP strongly interfere with the analysis, while the spectra collected from samples onto glass were characterized by a poor S/N ratio. The deposition onto aluminum, as verified also by Bedoni and coworkers [55], is rather correlated with well-defined Raman bands, which are easily associable to the vibrational signatures of several biomolecules. Figure 6 shows the comparison of Raman spectra acquired at 785 nm of saliva before (Figure 6a) and after (Figure 6b) filtering with 3 kDa filters.

The characteristic features of proteins are clearly recognizable in the spectra of both saliva and salivette, dominating the investigated spectral region. In the spectra obtained after the cut-off at 3 kDa, the only signals related to proteins are the out-of-ring breathing of tyrosine (824 cm^−1^), the C–C stretching of the proline ring (926 cm^−1^), the C–C stretching of the protein β-sheet (978 cm^−1^), and the band of Amide III (centered at 1255 cm^−1^). Saliva treatment with filters to remove large biomolecules is thus necessary in Raman spectroscopy to obtain information from smaller metabolites. Protein precipitation with EtOH, instead, gives Raman spectra with high noise and low-intensity signals, and no reliable information could be deduced from them.

The PCA was applied to the preprocessed dataset acquired at 785 nm, obtaining a 95.6% of variance explained by the first two PCs (Figures S4 and S5). Saliva and salivette spectra cluster together and are clearly separated from the other samples along PC2. It appears, thus, that the Salivette^®^ swab does not retain/release any compound at a significant concentration for Raman. The spectra of saliva_CO and salivette_CO are separated along PC1, while they appear indistinguishable along PC2, and a detailed analysis of the spectra revealed that salivette_CO samples show Raman signals at a lower intensity with respect to those of saliva_CO. As would be expected, the samples treated with EtOH form a close-packed cluster separated from the other groups.

Spectra acquisition with a laser in the visible range is further complicated by molecular fluorescence. Specifically, we could not register any Raman working at 532 nm regardless of the processing protocol, while at 633 nm, protein removal with 3 kDa filters was necessary. In this case, the spectra of saliva_CO and salivette_CO mostly resemble those acquired at 785 nm, though the spectral bands are broader and less defined.

## 4. Conclusions

Vibrational spectroscopy (ATR-FTIR and Raman) of saliva in tandem with chemometrics is potentially a straightforward technique for pathology biomarker research and for personalized medicine screening to facilitate the diagnosis and follow up of patients during pharmacological therapies once biomarkers have been identified.

Multivariate analysis suggests that both Raman and FTIR spectral patterns are not affected by the saliva collection method (spitting or swab). The deproteinization method, instead, may affect the results of saliva-based vibrational spectroscopy, most of all because saliva contains nonprotein nitrogen that precipitates in ethanol [54]. Thus, the collection–processing protocol should be based on the biochemical component suitable to obtain differential diagnoses or to extract information on specific biomarkers [4]. As for the other spectrochemical approaches, FTIR is in fact advantageous for providing holistic information, but the extraction of information from the spectra is a key point to make this information useful for clinical purposes.

Although saliva collection by cotton swabs is not invasive, the spitting/drooling method is even easier and minimizes patient hassle, and it is cost-effective in repeated “personal monitoring” when the dynamics of salivary metabolites would be required. Raman analysis before and after protein removal with cut-off filters allows to obtain complementary information. It is not trivial or negligible to highlight that the development of methods based on vibrational spectroscopies, coupled with easy preanalytical steps (sampling/processing) and portable infrared and Raman spectrophotometers would in principle favor bedside applications. Lastly, the saliva deposition of multiple spots onto low-cost PP sheets and the acquisition of spectra on “printed” microamounts of SDSs transferred onto ATR diamond window is fast and novel, and the samples dry simultaneously, and it allows to obtain reproducible conditions and spectra, even when small amounts of sample are available.

## Figures and Tables

**Table 1:** Sample preparation for FTIR analysis.

Application	Preanalytical Step	Ref.
COVID-19 Positive patients vs. controls	3 μL saliva (sampling not specified) on the ATR crystal and dried at RT for 15 min.	[28]
Screening Test for COVID-19	WS (sampling not specified) deposited onto a transflection substrate, dried (10 min), and analyzed by ATR.	[34]
COVID-19 Positive patients vs. controls	5 μL saliva (sampling not specified) on aluminum foil and air-dried at RT overnight.	[27]
Diabetic patients vs. controls	50 μL of unstimulated WS by expectoration dried under vacuum on BaF_2_ windows.	[35]
Diabetic patients vs. controls	3 μL saliva by spitting and dried at RT for 15 min on the ATR crystal.	[16,21,28]
Correlation FTIR spectra/surface tension; FTIR spectra/age and gender	50 μL WS (collected by spitting) on zinc selenide, dried at 37 °C for 60 min, and analyzed by ATR.	[36,37]
Burning mouth syndrome (BMS) vs. controls	30 μL WS (collected by spitting) on platinum, dried at 40 °C, and analyzed in diffuse reflectance mode.	[23]
Salivary gland tumor vs. controls	20 μL WS (collected by spitting) on zinc selenide, dried at RT, and analyzed by ATR.	[25]
Correlation FTIR spectra/biochemical composition	50 μL WS (collected by spitting) on zinc selenide, dried at 37 °C for 60 min, and analyzed by ATR.	[38]
Effects of saliva sample preparation	10 μL WS or saliva collected by spitting methods or cotton swab, dried as is or after centrifugation on germanium crystal or saliva concentrate after 4 h at 60 °C; analyzed by ATR.	[4]
Periodontitis vs. controls	50 μL WS collected by spitting, dried onto BaF_2_, and analyzed by transmittance FTIR.	[39]
Diabetes and periodontitis vs. controls	WS collected by spitting, dried, and analyzed onto ATR crystal.	[20]
Salivary profile of athletes	WS collected by spitting; 1.5 mg of dried saliva analyzed onto FTIR-ATR crystal.	[40]
Psoriasis status	30 μL WS collected by spitting deposited on a circular aluminum reflective surface, dried in hot air flow, and analyzed by transflectance FTIR.	[41]
Maximal Progressive Test in Athletes	100 μL WS collected by spitting, dried, and analyzed by ATR.	[42]
Folic Acid Deficient Pregnant Women vs. controls	WS collected by spitting deposited on TlBr crystal, dried, and analyzed by transmittance FTIR.	[43]
Physiological stress vs. controls	2 μL saliva (sampled by Salivette, Sarstedt) deposited on the ATR crystal, and dried at RT for 15 min.	[44]
Breast cancer patients vs. controls	Lyophilized saliva (sampled by Salivette, Sarstedt), dried, and analyzed by ATR crystal.	[24]
Oral Submucous Fibrosis (OSMF) vs. controls	3–5 μL saliva collected by Salivette, Sarstedt, dried, and analyzed by ATR.	[22]
Detection of SARS-CoV-2 Infection	WS collected by pharyngeal cotton swabs directly analyzed onto ATR crystal.	[26]

RT = room temperature; WS = whole saliva.

**Table 2:** Principal Mid-Infrared (MIR) Bands of the Dataset and Chemical Assignments [7,10].

MIR Frequency	Band Tentative Assignment
∼3736 cm^−1^	stretching O–H
∼3346 cm^−1^	stretching N–H
∼3275 cm^−1^	stretching O–H symmetric
∼3200−3550 cm^−1^	symmetric and asymmetric vibrations attributed to water
∼2968 cm^−1^	CH3 stretching
∼2930 cm^−1^	stretching C–H
∼2800−3000 cm^−1^	C–H lipid region
∼2100 cm^−1^	combination of hindered rotation and O–H bending (water)
∼1750 cm^−1^	lipids: ν(C=C)
∼1650 cm^−1^	amide I: ν(C=O)
∼1637 cm^−1^	H–O–H scissoring
∼1550 cm^−1^	amide II: δ(N–H) coupled to ν(C–N)
∼1450 cm^−1^	methyl groups of proteins: δ CH2 and CH3 asymmetric
∼1400 cm^−1^	methyl groups of proteins: δ CH2 and CH3 symmetric
∼1392–1396 cm^−1^	fatty acids and polysaccharides
∼1250−1260 cm^−1^	amide III: ν(C–N)
∼1155 cm^−1^	carbohydrates: ν(C–O)
∼1225 cm^−1^	DNA and RNA: νas(PO2−)
∼1080 cm^−1^	DNA and RNA: νs(PO2−)
∼1030 cm^−1^	glycogen vibration: νs(C−O)
∼992–986 cm^−1^	ribose phosphate main chain and stretching vibration C–C of DNA backbone
∼971 cm^−1^	nucleic acids and proteins: n(PO4)
∼960−966 cm^−1^	C–O, C–C, deoxyribose νs = symmetric stretching; νas = asymmetric stretching; and δ = bending.

## Data Availability

Data are available on request. Data is not publicly available due to privacy or ethical restrictions.

## Supplementary Materials

The following supporting information can be downloaded at: https://www.mdpi.com/article/10.3390/metabo13030393/s1, Figure S1. PCA results of SNV-normalized and centered (no scaling) ATR-FTIR spectra of saliva samples. (a) Score plot (69.6% of total variance); (b) loading plot PC1 (black line) and PC3 (green line). HMWsaliva_CO and HMWsalivette_CO refer to high-molecular-weight compounds (HMWCs) remaining in the upper part of 3 kDa cut-off filtering units (w = wiping, p = printing) as explained in the experimental part. Figure S2. PCA results of SNV-normalized and centered (no scaling) ATR-FTIR spectra of saliva samples. (a) Score plot (28.0% of total variance); (b) loading plot PC2 (red line) and PC3 (green line). HMWsaliva_CO and HMWsalivette_CO refer to high-molecular-weight compounds (HMWCs) remaining in the upper part of 3 kDa cut-off filtering units (w = wiping, p = printing) as explained in the experimental part. Figure S3. Absorbance HPLC chromatograms at 220 nm of saliva (a panel) and salivette (b panel) samples before and after deproteinization with 3 kDa cut-off filtration and precipitation in ethanol. (c) UV/visible spectra of the peak at tR = 5.2599 (purple line) and 4.35 min (blue line) of saliva_EtOH sample and tR = 5269 min (orange line) of saliva_CO sample. Figure S4. PC1 vs. PC2 score plots of Raman spectra acquired at 785 nm and preprocessed as described in Section 3.4. Legend: 1 (blue)—saliva; 2 (light blue)—salivette; 3 (green)—saliva_CO; 4 (yellow)—salivette_CO; 5 (orange)—saliva_EtOH; and 6 (red)—salivette_EtOH. Figure S5. PC1 (blue line) vs. PC2 (red line) loading plot of Raman spectra acquired at 785 nm and preprocessed as described in Section 3.4.
