# Supplementary material for: The Role of the Preanalytical Step for Human Saliva Analysis via Vibrational Spectroscopy
Source: Metabolites. 2023 Mar 8;13(3):393. doi: 10.3390/metabo13030393 (PMC10055013; doi:10.3390/metabo13030393)
Supplement: Supplementary file 1 [file metabolites-13-00393-s001.zip › metabolites-2243609-supplementary.pdf]

SUPPORTING INFORMATION TO

Article

The role of the pre-analytical step for human saliva analysis via vibrational spectroscopy

Beatrice Campanella [1], Stefano Legnaioli[1], Massimo Onor[1], Edoardo Benedetti [3] and Emilia Bramanti [1,*]

[1] National Research Council of Italy, C.N.R., Institute of Chemistry of Organometallic Compounds-ICCOM, 56124, Pisa, Italy

[3] Hematology Unit, Department of Oncology, University of Pisa, 56100 Pisa, Italy

* Correspondence: bramanti@pi.iccom.cnr.it; Tel.: +39-050-315-2293

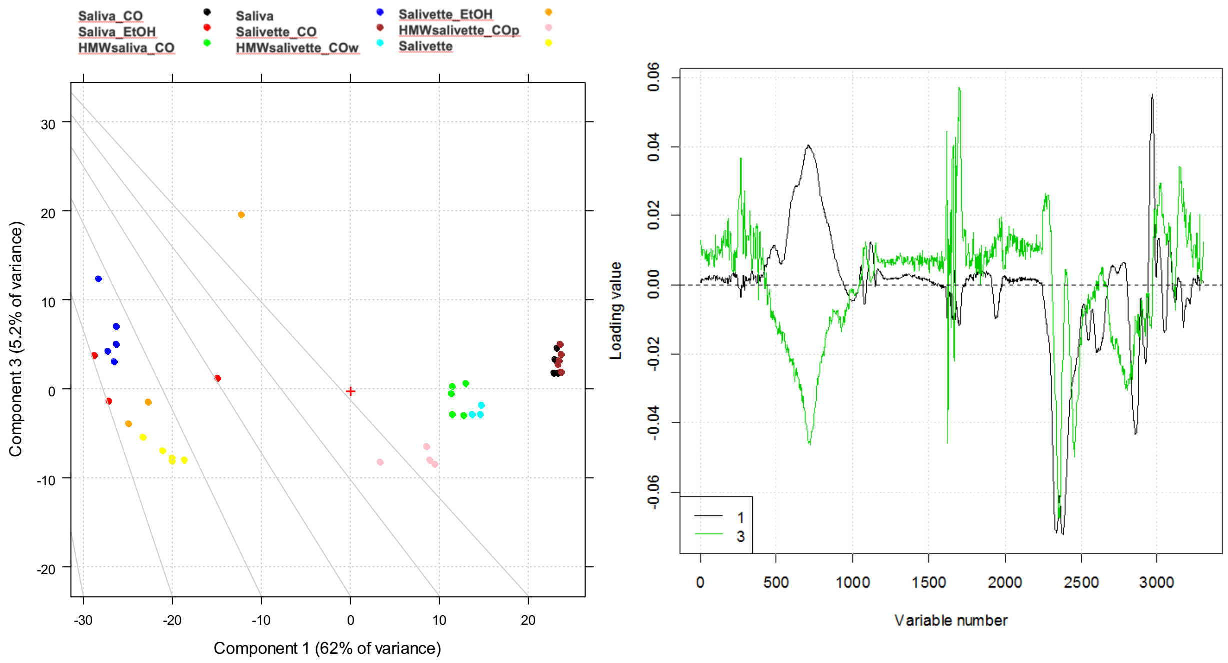

Figure S1. PCA results of SNV normalized and centered (no scaling) ATR-FTIR spectra of saliva samples. (a) Score plot (69.6% of total variance); (b) loading plot PC1 (black line) and PC3 (green line). HMWsaliva_CO and HMWsalivette_CO refer to high molecular weight compounds (HMW) remaining in the upper part of 3 kDa cut off filtering units (w=wiping, p=printing) as explained in the experimental part.

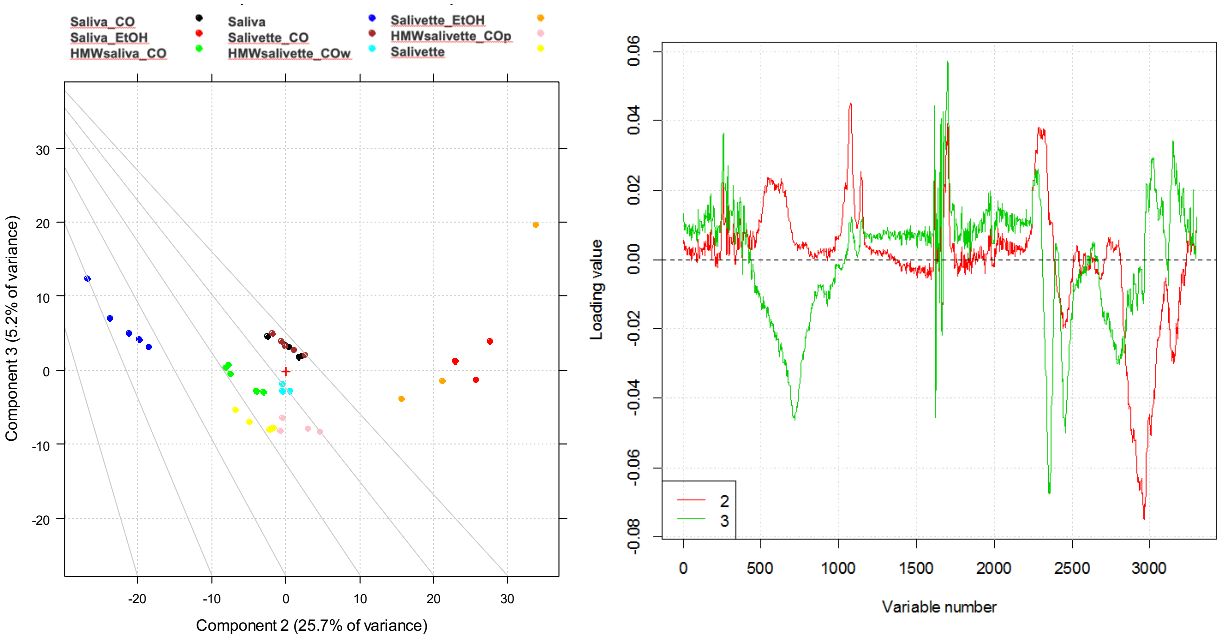

Figure S2. PCA results of SNV normalized and centered (no scaling) ATR-FTIR spectra of saliva samples. (a) Score plot (28.0% of total variance); (b) loading plot PC2 (red line) and PC3 (green line). HMWsaliva_CO and HMWsalivette_CO refer to high molecular weight compounds (HMW) re-maining in the upper part of 3 kDa cut off filtering units (w=wiping, p=printing) as explained in the experimental part.

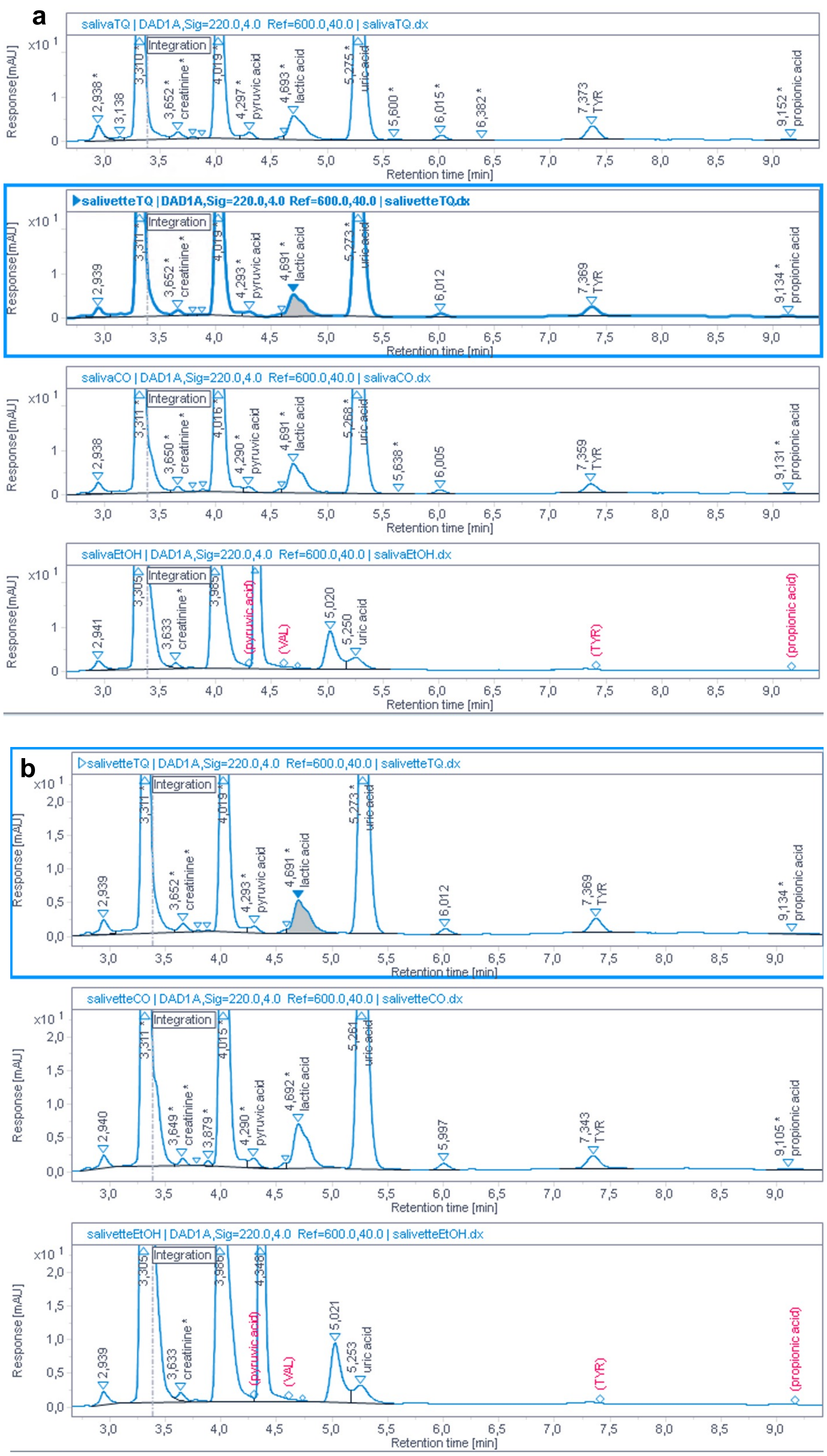

a
salivaTQ | DAD1A,Sig=220.0,4.0 Ref=600.0,40.0 | salivaTQ.dx
Integration
2,938 *
3,138
3,310 *
3,652 * creatinine *
4,019 *
4,297 * pyruvic acid
4,693 * lactic acid
5,275 * uric acid
5,600 *
6,015 *
6,382 *
7,373 TYR
9,152 * propionic acid
salivetteTQ | DAD1A,Sig=220.0,4.0 Ref=600.0,40.0 | salivetteTQ.dx
2,939
3,311 *
3,652 * creatinine *
4,019 *
4,293 * pyruvic acid
4,691 * lactic acid
5,273 * uric acid
6,012
7,369 TYR
9,134 * propionic acid
salivaCO | DAD1A,Sig=220.0,4.0 Ref=600.0,40.0 | salivaCO.dx
2,938
3,311 *
3,650 * creatinine *
4,016 *
4,290 * pyruvic acid
4,691 * lactic acid
5,268 * uric acid
5,638 *
6,005
7,359 TYR
9,131 * propionic acid
salivaEtOH | DAD1A,Sig=220.0,4.0 Ref=600.0,40.0 | salivaEtOH.dx
2,941
3,305
3,633 creatinine *
3,985
(pyruvic acid)
(VAL)
5,020
5,250 uric acid
(TYR)
(propionic acid)
b
salivetteTQ | DAD1A,Sig=220.0,4.0 Ref=600.0,40.0 | salivetteTQ.dx
2,939
3,311 *
3,652 * creatinine *
4,019 *
4,293 * pyruvic acid
4,691 * lactic acid
5,273 * uric acid
6,012
7,369 TYR
9,134 * propionic acid
salivetteCO | DAD1A,Sig=220.0,4.0 Ref=600.0,40.0 | salivetteCO.dx
2,940
3,311 *
3,649 * creatinine *
3,879 *
4,015 *
4,290 * pyruvic acid
4,692 * lactic acid
5,261 uric acid
5,997
7,343 TYR
9,105 * propionic acid
salivetteEtOH | DAD1A,Sig=220.0,4.0 Ref=600.0,40.0 | salivetteEtOH.dx
2,939
3,305
3,633 creatinine *
3,986
4,348
(pyruvic acid)
(VAL)
5,021
5,253 uric acid
(TYR)
(propionic acid)
Response [mAU]
x10 1
Retention time [min]

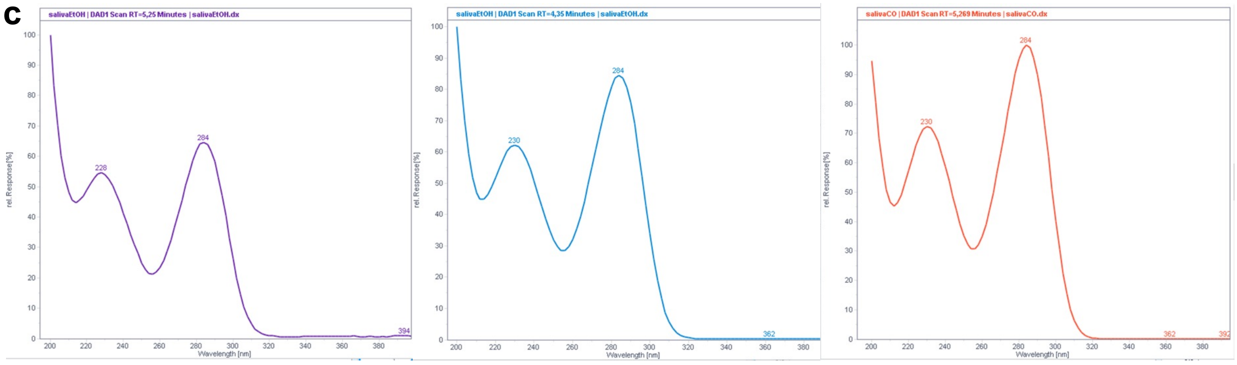

Figure S3. Absorbance HPLC chromatograms at 220 nm of saliva (a panel) and salivette (b panel) samples before and after deproteinization with 3 kDa cut off filtration and precipitation in ethanol. (c) UV/visible spectra of the peak at t_R=5.2599 (purple line) and 4.35 min (blue line) of saliva_EtOH sample and tR=5,269 min (orange line) of saliva_CO sample.

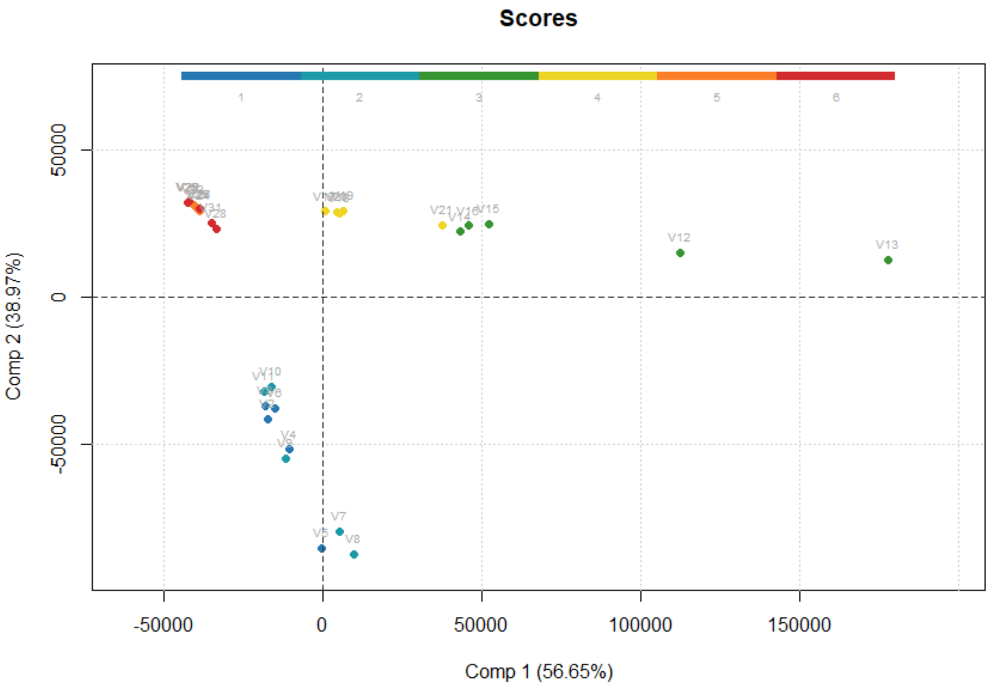

Figure S4. PC1 vs. PC2 score plot of Raman spectra acquired at 785 nm and preprocessed as described in Section 3.4. Legend: 1 (blue) – saliva; 2 (light blue)– salivette; 3 (green) – saliva_CO; 4 (yellow) – salivette_CO; 5 (orange)– saliva_EtOH; 6 (red) – salivette_EtOH.

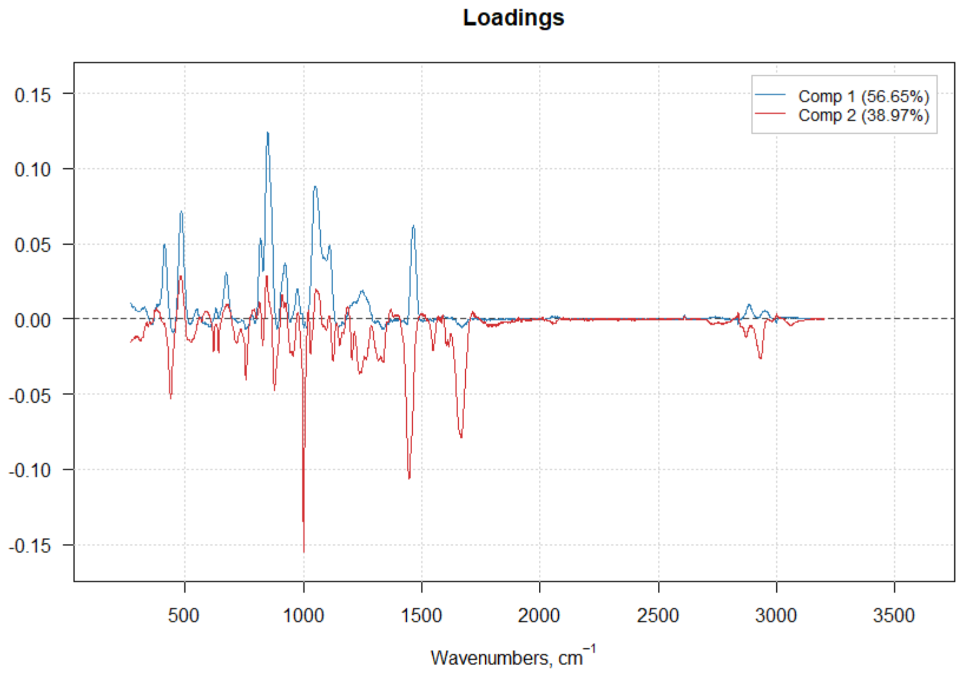

Figure S5. PC1 (blue line) vs. PC2 (red line) loading plot of Raman spectra acquired at 785 nm and preprocessed as described in Section 3.4.
